# Dr. Lewin, on the Use of Artificial Musk

**Published:** 1801-06

**Authors:** R. Lewin

**Affiliations:** Liverpool


### *To the Editors of the Medical and Phyfical Journal.*

Gentlemen,

WHILST the efficient caufes of many phenomena in nature remain veiled amidft impenetrable obfcurity, the philofophic mind is occafionally cheered with the illumination of a general principle prefented to his view by the laborious induction of facts. Hence, to apologife in prefenting a medical cafe or two, would to you be fuperfluous.

During the late feafon, when dyfentery raged with more than ufual violence, a very fimple treatment was found very fuccefsful; the oleum ricini, in the form of an emulfion, with the occafional ufe of anodynes, was my conftant practice. When ftrong strong inflammatory, or urgent typhoid symptoms supervened, the anti-phlogistic or tonic plan was superadded.

Under this simplicity of treatment, I had the gratification to see a numerous set of patients recover, without exception, except in two instances. The first was of a person who neglected to obtain medical aid till within a few hours of death; the other is one of the subjects of this communication.

After the resolute perseverance in the above plan during three weeks, James Houghton, ætat. 4, was sensibly and rapidly declining; his appetite was gone, his emaciation very considerable, his sleep much disturbed, his pain in the abdomen incessant, his pulse rapid and feeble, and his loss of blood per anum, from its quantity, highly alarming. In short, a gradual decline had apparently begun. Under these circumstances, I felt hurt at the idea of losing a patient in a disease whose malignity I had deemed, from experience, not to be dreaded. He was the only child of affectionate parents, hence an object of peculiar anxiety to a medical practitioner. Under these circumstances, I used the artificial musk mentioned in your Journal, as from its effects there stated, and its chemical history, it appeared to augur well. After one week's exhibition of this medicine, together with the continuance of laxatives, which were still indispensable, the child demanded animal food, but rejected vegetables; he was indulged, and a beef-steak was not only taken with impunity, but with manifest advantage. The pain had subsided, his sleep was refreshing, and his pulse, from this time, both as to velocity and strength, returned by gradual progression to its natural standard.

The other case with which I wish to trouble you, is of a boy seven years old, and afflicted with pertussis. The fits of coughing, together with the succeeding inhalation of breath, were always attended with a very near approximation to general convulsion. Pain of the side was acute, head severely affected, pulse rapid, feeble, and intermitting.

He had been previously attended, with much judgment, by one of the apothecaries to our Dispensary; yet the symptoms becoming daily worse, he applied to me.

His family I find to be consumptive; his aunt is now labouring under a severe hæmoptoe, and has a scrophulous aspect; his brother died of hooping cough, after long lingering in a similar state to the survivor. After the application of leeches, and a blister to the side, I attempted the use of Digitalis, which had been renounced before my attendance, owing to the stomach rejecting it. I was under the same necessity of yielding to similar circumstances. I now resorted to assafœtida. This is a medicine whose efficacy in the latter stage of this disease has been occasionally remarked to be very great, and at other times to be very inert.

The pain of the side was now removed, but other symptoms of a more threatening nature presented. His emaciation increased; morning sweats regularly prevailed; and pain, nearly constant, harrassed him in the lumber region. This last symptom was much aggravated by the fits of coughing.

A hectic flush in the afternoon, and expectoration of blood, began to shew themselves, and he complained of considerable chilliness. We had now recourse to the artificial musk. During a fortnight, the symptoms appeared to be stationary. The pain which I last mentioned, ceased about this time, and the pulse assumed a regular and fuller beat.

Since this period the morning sweats and threatened hectic have vanished; the countenance has now the cheerful playfulness of childhood; his strength is returning; his appetite is good, and the paroxysms of coughing are, comparatively, seldom, and mild. No other medicine was given at this time; we may, therefore, attribute this state of convalescence to the unassisted energy of this powerful remedy.

In cases of anorexia and general irritability of the stomach induced by the baneful excesses of inebriation, it will be found beneficial. I have only prescribed it to *spirit drinkers*.

Since the dysenteric case already recited, it has been advantageously used by adults when the disease has been long protracted.

My formula has been, the tincture joined with the dec. lin. so as to administer from fifteen to twenty or thirty drops to children, and even fifty to adults, twice or thrice within the twenty-four hours. I am, &c.

*Liverpool, April* 10, 1801. R. LEWIN.

